# Catecholaminergic Crisis After a Bleeding Complication of COVID-19 Infection: A Case Report

**DOI:** 10.3389/fendo.2021.693004

**Published:** 2021-09-08

**Authors:** Angel Rebollo-Román, Maria R. Alhambra-Expósito, Yiraldine Herrera-Martínez, F. Leiva-Cepas, Carlos Alzas, Concepcion Muñoz-Jiménez, R. Ortega-Salas, María J. Molina-Puertas, Maria A. Gálvez-Moreno, Aura D. Herrera-Martínez

**Affiliations:** ^1^Endocrinology and Nutrition Service, Reina Sofia University Hospital, Córdoba, Spain; ^2^Maimonides Institute for Biomedical Research of Córdoba, Córdoba, Spain; ^3^Nuclear Medicine Service, Virgen del Rocio University Hospital, Seville, Spain; ^4^Pathology Service, Reina Sofia University Hospital, Córdoba, Spain

**Keywords:** bleeding, pheochromocytoma, COVID19, catecholamines, complication

## Abstract

The severe acute respiratory syndrome coronavirus 2 (SARS-CoV-2) presents in some cases with hemostatic and thrombotic complications. Pheochromocytomas are unusual, though potentially lethal tumors. Herein we describe the first case of hemorrhage in a pheochromocytoma related to SARS-CoV-2 infection. A 62-year-old man consulted for syncope, fever, and palpitations. He was diagnosed with SARS-CoV-2 pneumonia and presented with a hemorrhage in a previously unknown adrenal mass, which resulted in a catecholaminergic crisis. Medical treatment and surgery were required for symptom control and stabilization. We hereby alert clinicians to watch for additional/unreported clinical manifestations in COVID-19 infection.

## Introduction

Pheochromocytomas are rare tumors derived from the adrenal medulla. Usually germline or somatic gene mutations are implicated, resulting in sporadic tumors, or associated with hereditary syndromes. These tumors might be diagnosed incidentally or due to clinical symptoms due to catecholamine overproduction or to a mass effect, but are rarely diagnosed because of intratumoral hemorrhage. Diagnosis is confirmed by elevated plasma/urine metanephrines or normetanephrines; additionally, imaging is necessary for tumor location and the evaluation of local invasion or metastases (1).

Coronavirus disease 2019 (COVID-19), caused by severe acute respiratory syndrome coronavirus 2 (SARS-CoV-2), has resulted in an emerging respiratory infection with pandemical diffusion since January 2020 (2). SARS-Cov-2 infection has been associated with several complications, including thrombosis and bleeding in comparable rates in patients with similar degrees of critical illness (3).

Although intratumoral hemorrhage in pheocromocytomas is a very rare manifestation of this tumor, some cases have been previously described, especially related with trauma or systemic anticoagulation (4–6); in most cases, any underlying cause was identified (7). In these patients, less than 30% had a previous history that suggested a pheochromocytoma (8, 9). Mortality rate in these cases reaches 28–31%, but lower rates should be currently expected due to early diagnosis and appropriate alpha blockage (7, 10).

It is suggested that paroxysms of hypertension or necrosis increase intratumoral intravascular pressure and may produce hemorrhage (11, 12). Trauma, thrombolysis, anticoagulants, or alpha-blockers could act as initiating factors (4–7, 10). Viral infections have not been previously described as precipitator of intratumor hemorrhage in pheocromocytomas.

Herein, we report, to the best of our knowledge, the first case of intratumoral hemorrhage of a pheochromocytoma in the context of a SARS-CoV-2 infection.

## Case Report

A 62-year-old patient presented to the emergency department with recurrent fainting episodes accompanied by asthenia and dry cough. Clinical symptoms appeared one week before consultation. The patient had a personal history of high blood pressure (HBP) treated with four drugs (losartan, hydrochlorothiazide, amlodipine, and furosemide). No previous history of bleeding or any other disease was described.

**Figure 1:**
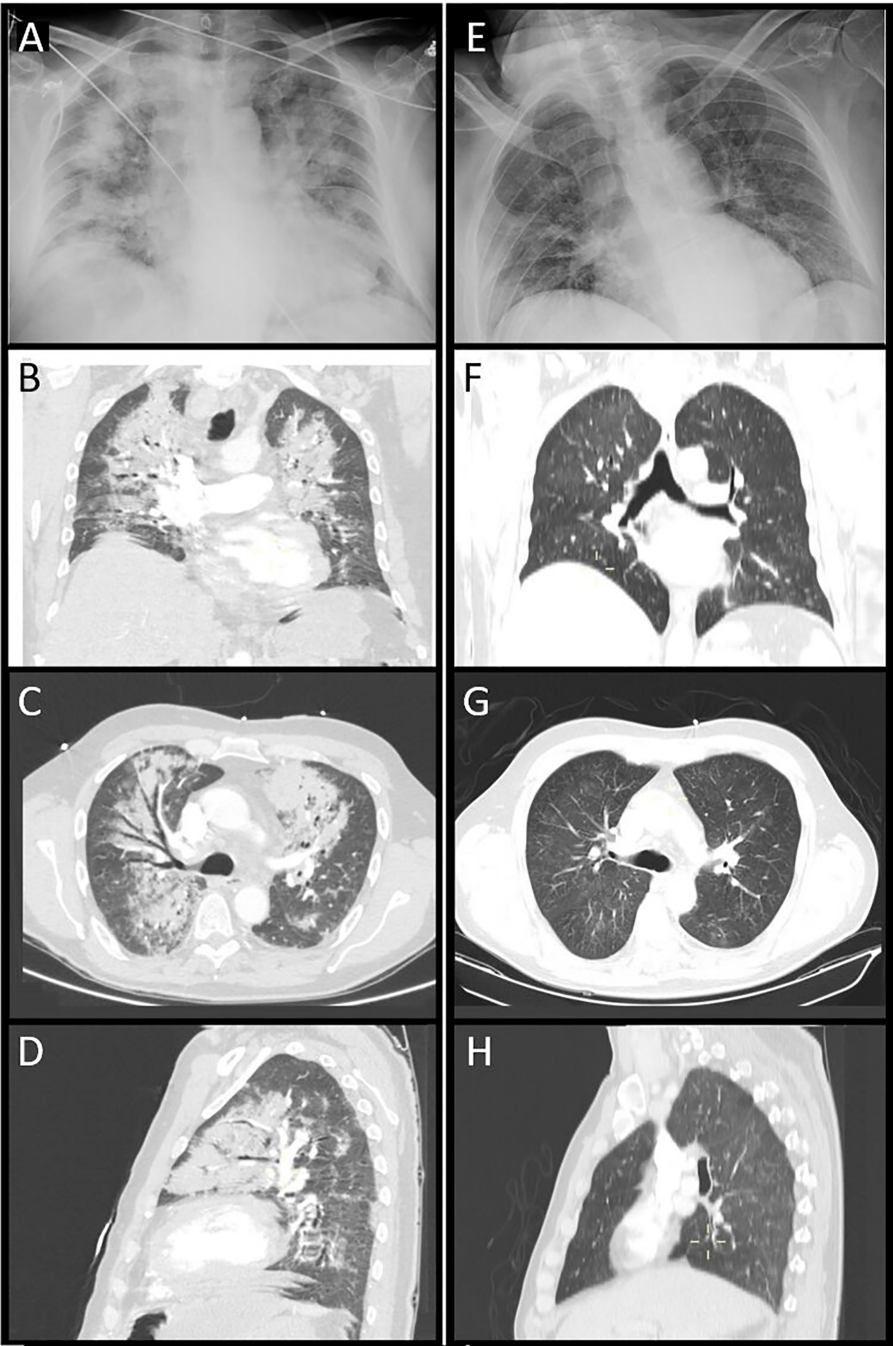
Thorax radiography **(A)** and CT images [coronal view, **(B)**; axial view, **(C)**; sagittal view, **(D)**]. Diffuse bilateral patched consolidations with open bronchi inside and ground-glass opacities due to COVID-19 infection. Thorax radiography **(E)** and CT control images. Two weeks after treatment [coronal view, **(F)**; axial view, **(G)**; sagittal view, **(H)**], significant improvement of the pneumonia is observed.

**Figure 2:**
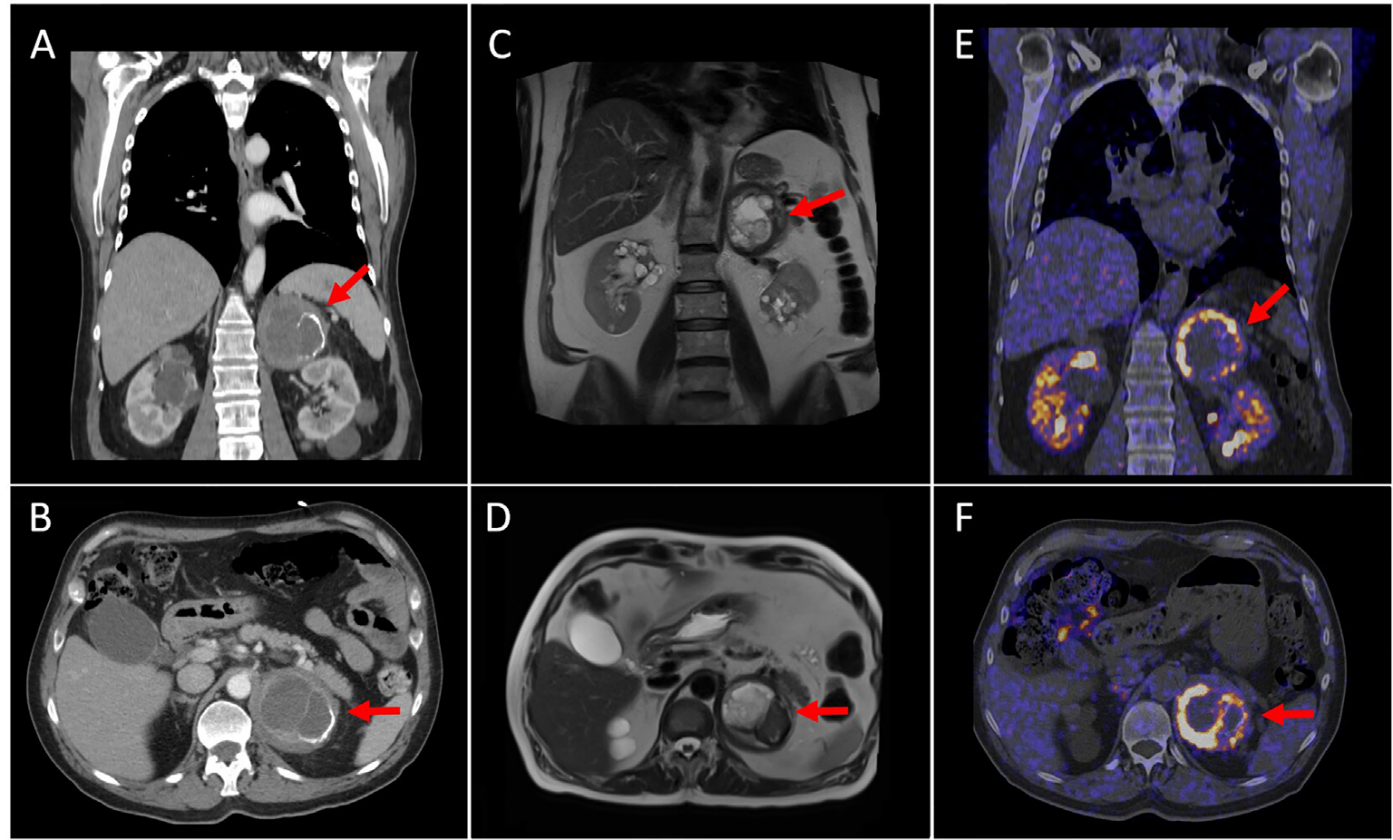
Coronal and axial CT views **(A, B)** show an 8 cm left adrenal tumor with a cystic component inside and partially calcified wall. Coronal and axial MRI views **(C, D)** reveal a left adrenal mass with a heterogeneous content (necrotic-cystic areas). Coronal and axial [^18^ F] DOPA PET/CT images **(E, F)** demonstrate intense and heterogeneous DOPA-uptake in the periphery of the mass (SUVmax of 20.9). These findings were compatible with the existence of pheochromocytoma with a cystic–necrotic–hemorrhagic component.

Upon his arrival, blood pressure was 97/52 mmHg with a heart rate of 100 bpm. The chest X-ray showed converging diffuse condensations in both pulmonary fields, predominantly central, suggestive of evolutioned SARS-CoV-2 infection (**Figure 1A**). Arterial blood gas analysis showed hypoxemia and hypocapnia; additionally, high white blood cell count with neutrophilia and elevated D-dimer were observed (**Table 1**). Given the low BP, the global respiratory insufficiency and a high suspicion of SARS-Cov-2 infection, a CT pulmonary angiogram was performed in order to rule out a pulmonary embolism. Bilateral patched consolidations with open bronchi inside and ground-glass opacities and no filling defects were observed in the pulmonary arteries (**Figures 1B–D**). The lower images of the scan revealed an adrenal left mass (8 × 7.4 cm) with a cystic component and a big calcification, suggestive of a hematoma or pseudocyst (**Figures 2A, B** **)**. These results were confirmed in a MRI (**Figures 2C, D** **)**.

**Table 1:** Laboratory parameters.

Parameter	Admission	Discharge
Hemoglobin	14.5 g/dl	8.5 g/dl
White blood cell count	19,350 WBC/mcL	13,390 WBC/mcL
C-reactive protein	166 mg/L	166 mg/L
pO2 (mmHg)	53	94
pCO2 (mmHg)	26	40
Plasma free metanephrines (N < 90 pg/ml)	350	<15
Plasma free normetanephrines (N < 180 pg/ml)	231	18
24-h urine fractionated metanephrines (N < 341 mcg/24 h)	4,032.6	–
24-h urine fractionated normetanephrines (N < 444 mcg/24 h)	1,990.34	–

N, normal.

Given the hemodynamic instability and the global respiratory insufficiency secondary to SARS-Cov-2 infection, the patient was admitted to the intensive care unit. Previous SARS-CoV-2 infection was confirmed using serological tests, nasopharyngeal swab, and bronchoalveolar lavage PCR. Two days after admission, respiratory symptoms improved but the patient remained hemodynamically unstable, alternating hypotension and hypertensive crises. Initially, intravenous treatment with noradrenaline 0.2 mcg/kg/min was administered but after 48 h, it was stopped.

**Figure 3:**
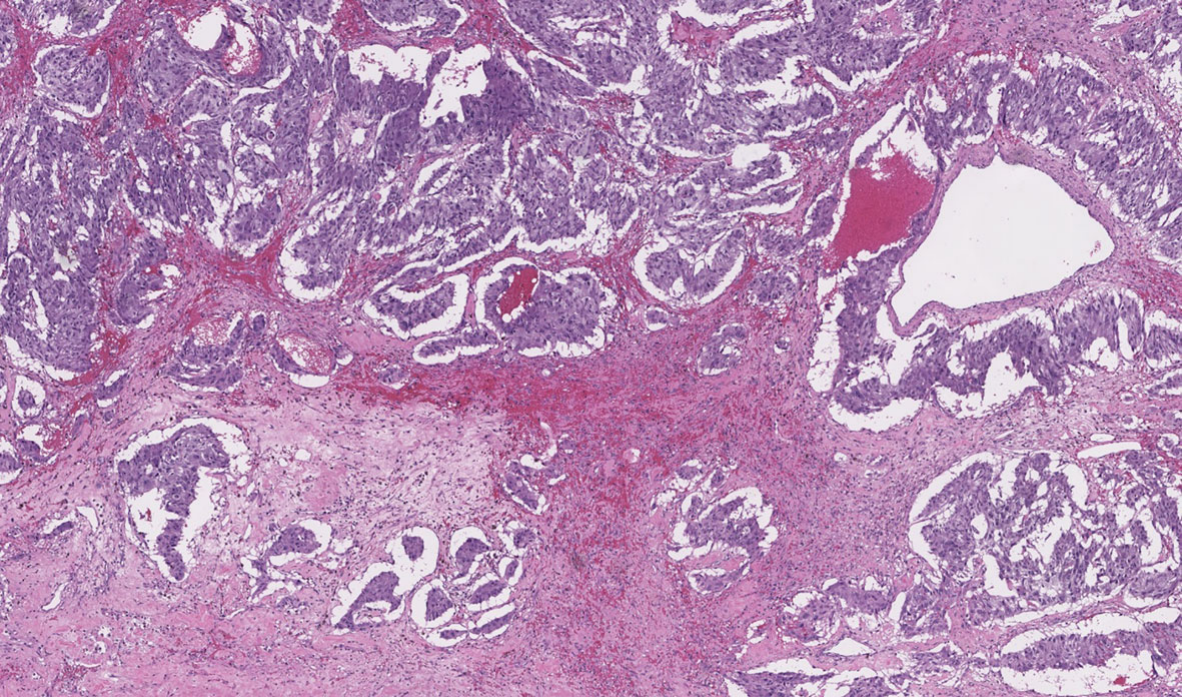
Histological characteristics of the resected tumor. Hematoxylin eosin images that show intratumor hemorrhage and 60% of necrosis without vascular or peritumoral invasion.

Elevated plasma and 24-h urinary metanephrine and normetanephrine were detected (**Table 1**). Simultaneously, hemoglobin levels dropped 2 g/dl. An 18-F-DOPA PET/CT was performed and revealed high aminoacidic metabolism in the peripheral area of the adrenal mass, with a central hypodense area with calcifications without metabolism, suggesting a pheochromocytoma with internal necrotic/cystic/hemorrhagic component (**Figures 2E, F** **)**. An open adrenalectomy with splenectomy was performed after adequate alpha- and beta-blockade using low-doses of doxazosin (2 mg/12 h) and bisoprolol (5 mg/d). The histological analysis reported a pseudoencapsulated pheochromocytoma of 7 × 5 × 4 cm and 340 g, with focal calcification, intratumor hemorrhage and 60% of necrosis without vascular or peritumoral invasion (**Figure 3**). Clinical and radiological improvements of the pneumonia were also observed (**Figures 1E–H**). A summary of biochemical parameters at admission and at discharged are depicted in **Table 1**. Eleven months after surgery the patient remains asymptomatic, without evidence of relapsed disease and requires any antihypertensive drugs.

## Discussion

SARS-Cov-2 infection has several clinical presentations, ranging from asymptomatic patients to mild symptoms and acute severe respiratory stress (2). Global mortality reaches 5.44% of cases, mostly related to respiratory insufficiency with hypoxia or multiple organ dysfunction (13), additionally some patients suffer severe systemic hyperinflammatory reaction (cytokine storm), which reminds that hemophagocytic lymphohistiocytosis is also triggered by other viral infections (5).

SARS-CoV-2 infection can be associated with coagulopathy alterations, probably due to infection-induced inflammatory changes, similar to those observed in patients with disseminated intravascular coagulation. Initially, coagulopathy presents with prominent elevation of D-dimer and fibrin/fibrinogen degradation products; in contrast, abnormalities in prothrombin time, partial thromboplastin time or platelet count are uncommon at a first stage of disease (3).

In this context, some cases of post-COVID19 spontaneous hemorrhage, including intracranial, pulmonary, abdominal, pelvic, and muscular hemorrhage, have been described (14). In some patients, this spontaneous hemorrhage has been reported as the presenting symptom of SARS-Cov-2 infection (15, 16). A multicenter, retrospective study performed in 400 hospital-admitted SARS-Cov-2 infected patients reported thrombocytopenia and decreased fibrinogen as clinical factors associated with significant bleeding manifestations (17); in this study, the overall bleeding rate was 4.8% (95% CI, 2.9–7.3%), specifically 3.1% (95% CI, 1.4–6.1%) in non-critically ill patients and 7.6% (95% CI, 3.9–13.3%) in critically ill patients. Remarkably, the major bleeding rate (WHO grade 3–4) was 2.3% (95% CI, 1.0–4.2%) (17), compared with the 5.6% rate observed in critically ill patients without SARS-Cov-2 infection and heparin thromboprophylaxis (18).

Furthermore, increased mortality rate has been described in patients with spontaneous intraabdominal hemorrhage; for example, a spontaneous hematoma in the ileo-psoas increases mortality rate by 28% in an intensive care unit (14). In this context, several institutions recommend personalizing the use of low molecular weight heparin or unfractionated heparin infusions in patients with SARS-Cov-2 infection (with elevated D-dimer levels and without known thrombotic complications), since the risk of hemorrhage is also present in these patients (19).

Herein we report a case with probable coagulation disturbances after a SARS-Cov-2 infection, which provoked adrenal mass bleeding and consequently catecholamine liberation; as a result, a catecholaminergic crisis was observed in this patient with incidental pheochromocytoma. Importantly, acute hemorrhage represents a differential diagnosis in this patient, but acute hemorrhagic rupture as the initial manifestation of pheochromocytoma is rare (10), and might be related with increased intratumoral intravascular pressure that may be precipitated by paroxysms of hypertension or necrosis (11, 20).

In our patient, D-dimer was elevated at the moment of his arrival. During the early phase of SARS-CoV-2 infection, coagulation test abnormalities are seen, but they do not result in clinical bleeding. Whether the initial coagulation changes seen in infected patients progress linearly to sepsis-induced coagulopathy and then to disseminated intravascular coagulopathy as a result of SARS-CoV-2 infection is still to be determined (3). Currently, it is not known whether the underlying cause of the elevated D-dimer levels, bleeding, and thrombotic manifestations in the SARS-Cov-2 infection are related to a pathophysiological-distinct viral coagulopathy or a coagulation system activation due to severe inflammation (17).

In summary, to the best of our knowledge, this is the first case report of adrenal hemorrhage in a pheochromocytoma related to SARS-CoV-2. Based on the current short experience and the chronological association, SARS-CoV-2 may be considered accountable for the hemorrhage in this patient.

## Data Availability Statement

The raw data supporting the conclusions of this article will be made available by the authors, without undue reservation.

## Ethics Statement

Written informed consent was obtained from the individual(s) for the publication of any potentially identifiable images or data included in this article.

## Author Contributions

All authors have equally contributed to clinical follow-up of the patient and the preparation of this manuscript. All authors contributed to the article and approved the submitted version.

## Funding

This work was funded by Instituto de Salud Carlos III, co-funded by European Union (ISCIII-AES-2019/002525). Abbott Nutrition kindly contributed with the publication fee of this article. The funder was not involved in the study design, collection, analysis, interpretation of data, the writing of the manuscript or the decision to submit it for publication.

## Conflict of Interest

The authors declare that the research was conducted in the absence of any commercial or financial relationships that could be construed as a potential conflict of interest.

## Publisher’s Note

All claims expressed in this article are solely those of the authors and do not necessarily represent those of their affiliated organizations, or those of the publisher, the editors and the reviewers. Any product that may be evaluated in this article, or claim that may be made by its manufacturer, is not guaranteed or endorsed by the publisher.

## Abbreviations

SARS-CoV-2, severe acute respiratory syndrome coronavirus 2; COVID-19, Coronavirus disease 2019; HBP, high blood pressure; CT, computed tomography; PCR, polymerase chain reaction; [^18^ F] DOPA-PET, 6-fluoro-(^18^F)-L-3,4-dihydroxyphenylalanine positron emission tomography; MRI, magnetic resonance imaging.
